# Efficient antibacterial AIEgens induced ROS for selective photodynamic treatment of bacterial keratitis

**DOI:** 10.3389/fchem.2022.1088935

**Published:** 2023-01-04

**Authors:** Wenting Cai, Tianyi Shen, Dong Wang, Tingting Li, Jing Yu, Chen Peng, Ben Zhong Tang

**Affiliations:** ^1^ Department of Ophthalmology, Shanghai Tenth People’s Hospital, School of Medicine, Tongji University, Shanghai, China; ^2^ College of Materials Science and Engineering, Shenzhen University, Shenzhen, China; ^3^ Department of Radiology, Shanghai Public Health Clinical Center, Fudan University, Shanghai, China; ^4^ Shenzhen Institute of Molecular Aggregate Science and Engineering, School of Science and Engineering, The Chinese University of Hong Kong, Shenzhen, Hong Kong SAR, China

**Keywords:** aggregation-induced emission (AIE), bacterial keratitis, *Staphylococcus aureus*, antimicrobial materials, photodynamic therapy

## Abstract

Bacterial keratitis (BK) is an acute infection of the cornea, accompanied by uneven epithelium boundaries with stromal ulceration, potentially resulting in vision loss. Topical antibiotic is the regular treatment for BK. However, the incidence rate of multidrug-resistant bacteria limits the application of traditional antibiotics. Therefore, a cationic aggregation-induced emission luminogens (AIEgens) named TTVP is utilized for the treatment of BK. TTVP showed no obvious cytotoxicity in maintaining the normal cell morphology and viability under a limited concentration, and revealed the ability to selectively combine with bacteria in normal ocular environment. After light irradiation, TTVP produced reactive oxygen species (ROS), thus exerting efficient antibacterial ability *in vitro*. What’s more, in rat models of *Staphylococcus aureus* (*S. aureus*) infection, the therapeutic intervention of TTVP lessens the degree of corneal opacity and inflammatory infiltration, limiting the spread of inflammation. Besides, TTVP manifested superior antibacterial efficacy than levofloxacin in acute BK, endowing its better vision salvage ability than conventional method. This research demonstrates the efficacy and advantages of TTVP as a photodynamic drug in the treatment of BK and represents its promise in clinical application of ocular infections.

## 1 Introduction

Bacterial keratitis (BK) is the sixth leading cause of global vision loss, accounting for 3.2% of the 36 × 10^6^ cases in both developing and developed countries, which may be associated with trauma, contact lens wear, ocular surgery, or deficiency of corneal sensation (Davis et al., 2020; Khan et al., 2020; Blindness, 2021). BK is characterized by epithelial defect with corneal stromal inflammation and ulceration. As the progression of BK or without timely and appropriate treatment, corneal abscess, stromal lysis and corneal perforation may lead to severe visual loss ultimately (Han et al., 2020; Tuft et al., 2022). Among bacterial pathogens, *Staphylococcus aureus* (*S.aureus*) is the predominant pathogen inducing keratitis since it is a common inhabitant on ocular surface (Silva et al., 2019; Zhang et al., 2019). The cornea of rats infected by *S. aureus* was therefore widely utilized to perform the experimental keratitis research (Zhang et al., 2021; Zhang et al., 2022b). At present, BK is commonly administrated with topical eye drops of broad-spectrum antibiotics such as levofloxacin (LVFX), ciprofloxacin and moxifloxacin (Li et al., 2020a). The overuse and misuse of antibiotics are the most important determinant for the occurrence of antibiotics resistance, even with super bacteria occurred (Zhou et al., 2020). In BK, the prognosis of the disease is severely affected by an increasing resistant trend towards widely used empirical antibiotics, such as combinations of cephalosporin or glycopeptide and aminoglycoside (Ung et al., 2019). Besides, norfloxacin was reported to mediate apoptosis by injuring mitochondrial transmembrane and activating death proteins, and eventually leading to necroptosis through RIPK1-RIPK3-MLKL pathway (Yang et al., 2020). In this regard, there is still an urgent need to explore new targets with low incidence of drug-resistance. Therefore, the development of alternative effective antibacterial strategy is urgently needed, which favors the reduction of emergency of the resistance of bacteria.

In response to such a severe situation of drug resistance, sterilization material based on light-to-heat conversion, photocatalysis, chemocatalysis and biological extraction were performed (Huo et al., 2021; Wang et al., 2022). Among them, photodynamic therapy (PDT) utilized reactive oxygen species (ROS) generation from photosensitizers (PSs) activated with light irradiation, which has been regarded as a vital strategy to deal with drug-resistant bacterial infections under the background of post-antibiotic era (Chen et al., 2019; Qi et al., 2019; Liu et al., 2021; Zhou et al., 2021). The generation of abundant ROS could destroy bacterial cell membrane, inducing necrosis and apoptosis of bacteria (Idris et al., 2012; Han et al., 2016). Several studies have reported that PDT performed excellent anti-bacterial ability in the treatment of periodontitis (Li et al., 2021b), diabetic wound healing (Huang et al., 2021), malignant tumor (Cheng et al., 2020), immunomodulatory effects (Ayaz et al., 2020), rheumatoid arthritis (Lu et al., 2018) and bacterial ocular infection (Peng et al., 2021; Li et al., 2022). Encouragingly, PDT has advantage of excellent light transmission property, which is appropriate in the application of ocular infectious therapy (Fan et al., 2021). Thus, PDT with good antibacterial ability and biocompatibility showed a promising potential in the treatment of ocular infectious diseases. However, traditional PSs suffer from fluorescence quenching under irradiation, and generate low ROS due to π-π stacking in the aggregate state, which severely hindered their development of practical application (Liu et al., 2013; Jiang et al., 2018)**.**

Recently, luminogens with aggregation-induced emission (AIEgens) features showed broad application prospects for theranostic applications as PSs (Hu et al., 2018; Kang et al., 2019; Dai et al., 2020; Abrahamse et al., 2022). A delightful phenomenon could be observed that AIEgens exhibited excellent fluorescence and effective ROS creation during aggregation state while showing poor emission in the monomer state, which was resulting from the restriction of intramolecular motions (RIMs) (Mei et al., 2015; Wang et al., 2018a). AIEgens performed promising characteristics of high-efficiency of ROS production, low-toxicity and high-specificity targets (Li et al., 2020b). In addition, AIEgens have been broadly applied in fluorescent bioimaging due to splendid emission efficiency and large stokes shift (Yu et al., 2017). AIEgens have been reported to possess excellent biological applications, including granulomas tracking and targeted theranostics for tuberculosis (Liao et al., 2020), specific bacterial clearance and tumor elimination (Li et al., 2020b), and accelerated reduction of refractory internalized bacteria such as methicillin-resistant *Staphylococcus aureus* (MRSA) (Ni et al., 2020; Li et al., 2021a). Also, Hu et al. reported that M1-DPAN showed specific AIE ability, along with outstanding photostability and biosecurity, which take advantage of visualizing the occurrence of bacterial infection. Besides, it has been reported that AIEgens could selectively identify and aim at bacteria because of cationic charge and appropriate hydrophobicity, and exhibited vision defense *via* activating early intraocular immune response for bacterial endophthalmitis (Li et al., 2022). Such selective discrimination was attributed to the differences in membrane potential between bacteria and mammals (Yuan et al., 2014). Inspired by the previous studies, the design of cationic AIEgens, which performed selectively elimination of bacteria over normal cells is feasible.

**SCHEME 1:**
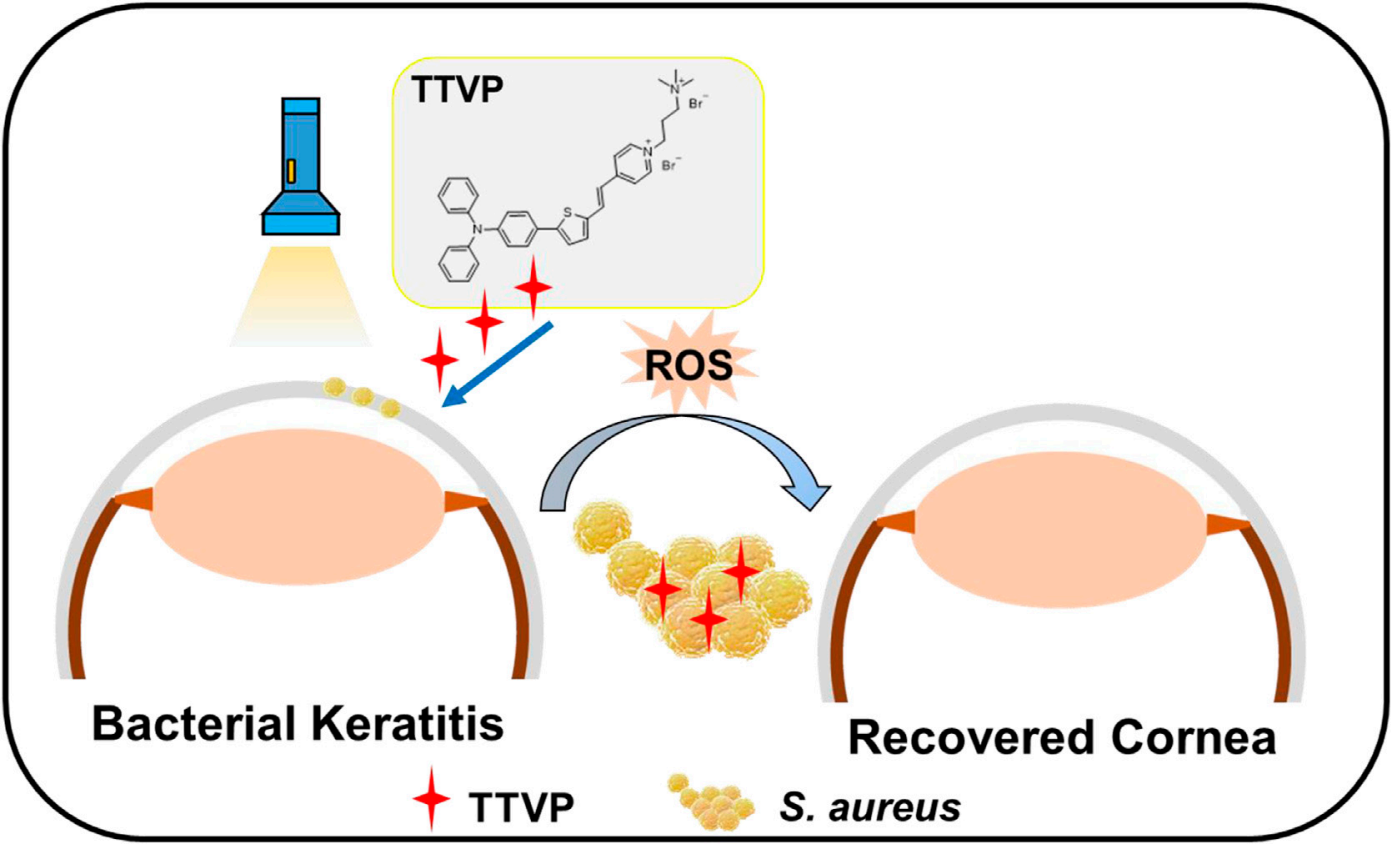
Schematic illustration of AIEgen TTVP for efficient photodynamic antibacterial therapy of BK.

In this study, we designed and synthesized a cationic AIEgens named TTVP, possessing the ability of identification and photodynamic antibacterial of Gram-positive bacteria (Lee et al., 2020), for the therapeutic schedule of bacterial keratitis. For the predominant position of *S. aureus* in pathogen of keratitis, we work on *S. aureus* to evaluate the killing efficiency of TTVP both *in vitro* and *in vivo* (Scheme 1). First of all, TTVP exhibited no obvious cytotoxicity in maintaining the normal cell morphology and viability under a limited concentration, and selectively combined with bacteria rather than normal ocular cells, which confirmed its biosafety in our following research. Under white light irradiation, TTVP generated abundant ROS production, leading to the death of *S. aureus*. The excellent killing efficiency of TTVP to *S. aureus* was further assessed *via in vitro* antibacterial activity studies. Furthermore, TTVP was applied to treat *S. aureus*-induced keratitis on a rat model, and excitingly, the infection recovery treated with TTVP was faster than that with LVFX *in vivo*. Attracted by these results, the outstanding treatment efficiency and good biosafety make TTVP promising for the clinical treatment of bacterial keratitis.

## 2 Experimental section

### 2.1 Materials and reagents

TTVP was synthesized in compling with the literature mentioned (Wang et al., 2018b). Other chemicals and reagents were bought from J&K Scientific and Sigma-Aldrich. DMEM/F12 cell medium, penicillin, streptomycin and fetal bovine serum (FBS) were obtained from HyClone (United States). Yeasen (Shanghai, China) provided cell counting kit (CCK)-8. *S. aureus* was acquired from China General Microbiological Culture Collection Center. SD rats were purchased from Beijing Vital River Laboratory Animal Technology Co., Ltd. (Beijing, China).

### 2.2 Characterization

Mother solution (5 mM) of TTVP was obtained through dimethyl sulfoxide (DMSO) initially, and then mixed with PBS to certain concentration. The aqueous dispersibility of TTVP in water, PBS and DMEM at the concentration of 5 μM was displayed excellently (Supplementary Figure S1). Ultraviolet-visible (UV-Vis) absorption and fluorescence (FL) spectra were measured by the Perkin Elmer Lambda 25 spectrophotometer and fluorescence spectrometry (Agilent Cary Eclipse), respectively.

### 2.3 Preparation of bacterial solution

A single *S. aureus* colony on solid agar plate was added to 10 ml liquid Luria broth (LB) culture medium. After incubation at 37°C overnight at 220 rpm, the bacteria were gathered by centrifugation (9,000 rpm, 3 min) and washed twice. After being resuspended with sterile normal saline, the concentrations were measured at 600 nm (1.0 OD = 10^8^ CFU ml^−1^). Finally, the *S. aureus* suspensions were diluted with PBS for further use.

### 2.4 *In vitro* antibacterial analysis

A standard plate counting method was carried out to evaluate sterilization effect of TTVP *in vitro* at concentrations of 0, 0.02, 0.05, 0.1 and 0.2 μM. The mixture was exposed to white light lamp for certain min (5, 10, 15 and 30 min), while the control group was exposed in dark. The treated bacterial suspension was diluted (1:5000) and incubated on the agar plate at 37°C for 24 h, followed by quantification and photo taking.

### 2.5 Assay for reactive oxygen species

ROS accumulation of TTVP was assessed According to a given standard protocol. The mixture of TTVP (0.1 μM) and DCFH-DA was incubated under a white light lamp (20 mW/cm^2^ power) for 15 min. The fluorescence intensity was recorded *via* a microplate reader before irradiation and every 1 min interval for 16 times (λex = 480 nm, λem = 525 nm). Meanwhile, OD values of DCFH-DA solution and PBS were recorded as control.

### 2.6 Zeta potential measurement

*S. aureus* was collected and resuspended with PBS, followed by incubating with different concentrations of TTVP (0, 0.02, 0.05, 0.1 and 0.2 μM) for 15 min. Then Zeta potential was measured *via* a Malvern Zetasizer.

### 2.7 Co-culture of bacteria and cells

*S. aureus* was co-cultured with human corneal epithelial cells (HCECs) to test the selective affinity of TTVP against bacteria. *S. aureus* suspension was added to pre-cultured adherent HCECs, along with different concentrations (0, 0.02, 0.05, 0.1 μM) of TTVP for 15 min. The slides were sealed and photographed using a fluorescence microscope.

### 2.8 *In vitro* cytotoxicity

The possible cytotoxicity of TTVP is a crucial issue to be examined before application. In general, cell viability, cell morphology and live/dead staining were performed to evaluate the biosafety of TTVP *in vitro*. In order to explore the biosafety of TTVP in keratopathy, research based on normal cells of ocular surface was necessary. Hence, HCECs were selected to evaluate the toxicity of TTVP.

Adherent HCECs were cultured in 96-well plates, being divided into irradiation group (20 mW/cm^2^, group a) and dark group (group b). After co-cultured with TTVP (0–1.0 μM) for 15 min, HCECs were placed in light or dark environment during the following 15 min. HCECs were continued culturing in the incubator for 24 h after removal of TTVP. Also, the cells were incubated for 24 h at the same concentration of TTVP in dark (group c) to detect the intrinsic cytotoxicity. Afterward, CCK-8 reagent was incubated for 2 h to obtain the fluorescence intensity at 450 nm. In addition, the cellular morphology of HCECs after incubation for 24 h was also recorded *via* optical microscopy.

For the live/dead staining analysis, HCECs were intervened with TTVP (0, 0.05, 0.1, 0.2 μM) for 24 h. Live and dead cells were labeled with calcein AM and PI, which excited green and red fluorescence under microscope, respectively.

### 2.9 Hemolysis assay

The hemocompatibility of TTVP was detected through hemolysis assay. Saline was added to the whole blood to wash several times until the supernatant was colorless. After diluting with saline to 2% red cell suspension, different TTVP concentrations (0, 0.02, 0.05, 0.1, and 0.2 μM) were added to the above red cell suspension, and heated at 37°C for 1 h. After centrifugation (3,000 rpm, 5 min), the images were captured and the ratio of hemolysis was calculated by the absorbance of supernatants at 540 nm recorded by a microplate reader. The erythrocytes treated with distilled water were regarded as positive control.

### 2.10 Animal experiments of antibacterial test

#### 2.10.1 *In vivo* antibacterial test on infected keratitis

The animal procedures involved were in accordance with the Association for Research in Vision and Ophthalmology Statement of Shanghai Tenth People’s Hospital. Six weeks of SD male rats were used to simulate the occurrence of BK as previously described with minor adjustments (Josyula et al., 2021). After general anesthetization by chloraldurate, 0.5% proparacaine hydrochloride ophthalmic solution was performed for local anesthesia. Prior to any operation, the images of rat corneas were captured. The operative eye was then scratched using a blade, and then *S. aureus* suspension (50 μl, 1 × 10^8^ CFU/ml) was placed over the ocular surface.

After being inoculated for 24 h, the rats were classified into four groups: 1) *S. aureus*-infected eyes treated by PBS, 2) *S. aureus*-infected eyes treated by TTVP under dark condition, 3) *S. aureus*-infected eyes treated by TTVP with light irradiation (20 mW/cm^2^ power, 15 min), and 4) *S. aureus*-infected eyes treated by LVFX. Photographs of the ocular surface were captured before treatment, as well as 1, 3 and 7 days after intervention to observe the infection and inflammation. The clinical inflammatory scores were calculated to qualify the severity. Seven days after treatment, eyeballs were fixed for H&E staining, Masson staining and immunohistochemistry staining analysis, or homogenized for spread plate assay.

#### 2.10.2 Clinical inflammatory scores

Clinical inflammatory scores of anterior segments, including size of corneal opacity area (grades 1–4, less than 25% to more than 75%), opacity density of cornea (grades 1–4, slightly misty to dense opacity) and hypopyon (grades 1 and 2, paracentral cornea involved or not) were performed to evaluate the severity of BK as previously reported (Zhang et al., 2019). Thus, the score of normal cornea was 0, while that of different groups was between 0 and 10.

#### 2.10.3 Spread plate test

The isolated corneas were homogenized in 0.5 ml of sterile PBS, diluted and cultured on agar plates for 24 h, followed by quantification and photo taking.

#### 2.10.4 HE, Masson and immunohistochemistry staining analysis

Eyeballs were fixed 7 days after infection for H&E and Masson staining to describe the bacterial histopathological reaction. Meanwhile, tumor necrosis factor-α (TNF-α) is the common primary inflammatory protein. Immunohistochemical staining of TNF-α was performed to display the inflammation at the site of the infected corneal lesions at 7 d follow-up. All photos were collected under the optical microscope.

### 2.11 *In vivo* biosafety

#### 2.11.1 Organ structure

At the end time points, the rats were sacrificed to harvest the heart, spleen, kidney, liver and lung for histopathology *in vivo*. After being fixed overnight, the tissues were embedded, sectioned, and stained with H&E, following standardized protocol. Neutral gum was applied to immobilize the sections, then the slides were imaged under an optical microscope.

#### 2.11.2 Serological assay

Whole blood of rats was reversed and centrifuged to obtain serum. Functions were measured according to the instructions. Liver and kidney functions including alanine aminotransferase (ALT) and aspartate aminotransferase (AST) and creatinine (CRE) were measured. Total cholesterol (T-CHO) and triglyceride (TG) reflected blood lipids, while potassium (K^+^), calcium (Ca^2+^), and chlorine (Cl^-^) represented electrolytic levels.

### 2.12 Statistical analysis

SPSS 23.0 software and GraphPad Prism 5 were utilized for statistical analyses. ImageJ version 1.48 was used for quantification of bacterial colonies. One-way ANOVA was carried out between multiple groups. *p* < 0.05 were identified as statistically significant.

## 3 Results and discussions

### 3.1 Optical property

**FIGURE 1:**
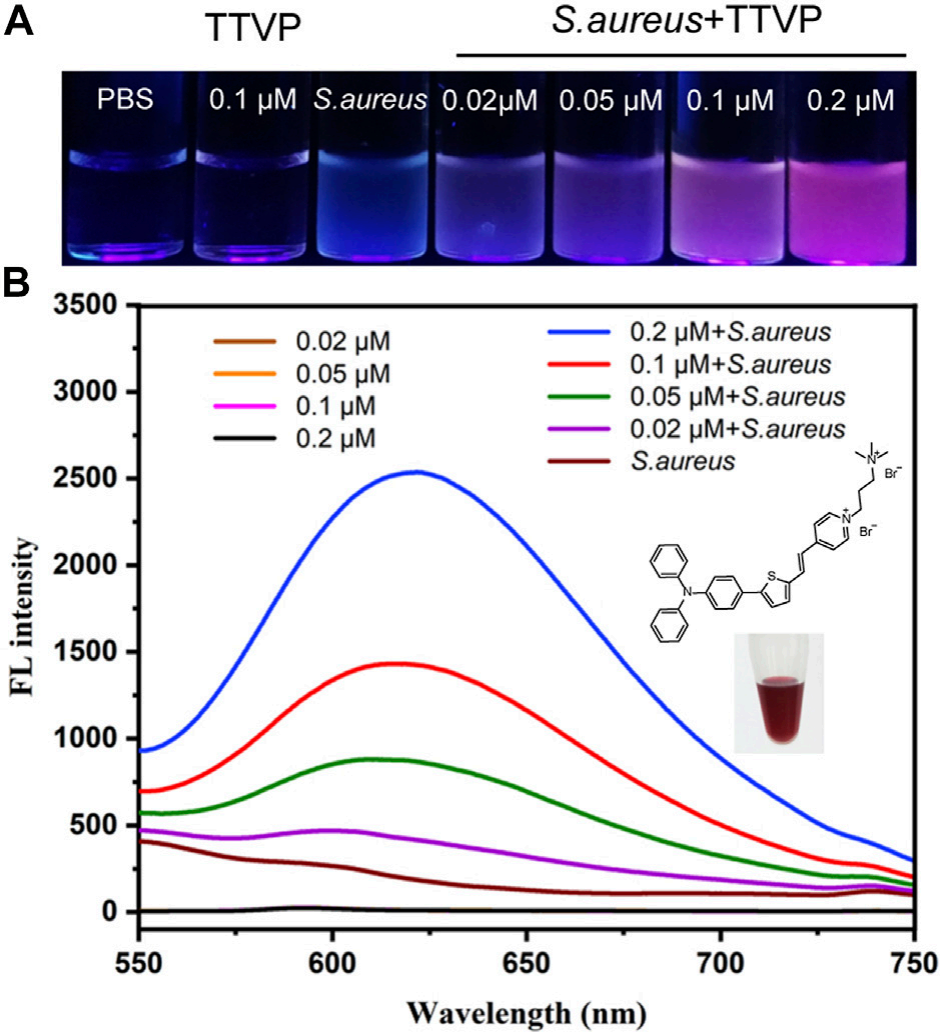
**(A)** The picture of *S. aureus* incubated with TTVP at different concentrations for 15 min under 365 nm UV light. **(B)** FL spectra of TTVP with or without incubation with *S. aureus* for 15 min (Excitation wavelength = 489 nm). The insert was the photograph of TTVP solution (5 mM) dissolved in DMSO.

The synthesis and purification of TTVP were under the guidance to the previous study (Wang et al., 2018b). As represented in Figure 1, UV—Vis and FL spectra were performed to verifythe optical characteristics of TTVP. After incubating TTVP in *S. aureus* for 15 min, red fluorescence was obviously visible under 365 nm UV irradiation, indicating that TTVP had a remarkable bioimaging effect on *S. aureus* (Figure 1A). Afterward, as shown in Figure 1B, TTVP solutions alone photoexcited by 489 nm showed no obvious difference in FL intensity under different concentrations. After a mixture with *S. aureus* at room temperature for 15 min, FL intensity showed the emission maximum wavelength at around 620 nm under concentration-dependent manner. The phenomenon might be due to the change of TTVP aggregation states of TTVP in response to *S. aureus*. TTVP has a strong affinity for *S. aureus* and may become a valuable visualization reagent for bacterial research.

### 3.2 *In vitro* antibacterial test

**FIGURE 2:**
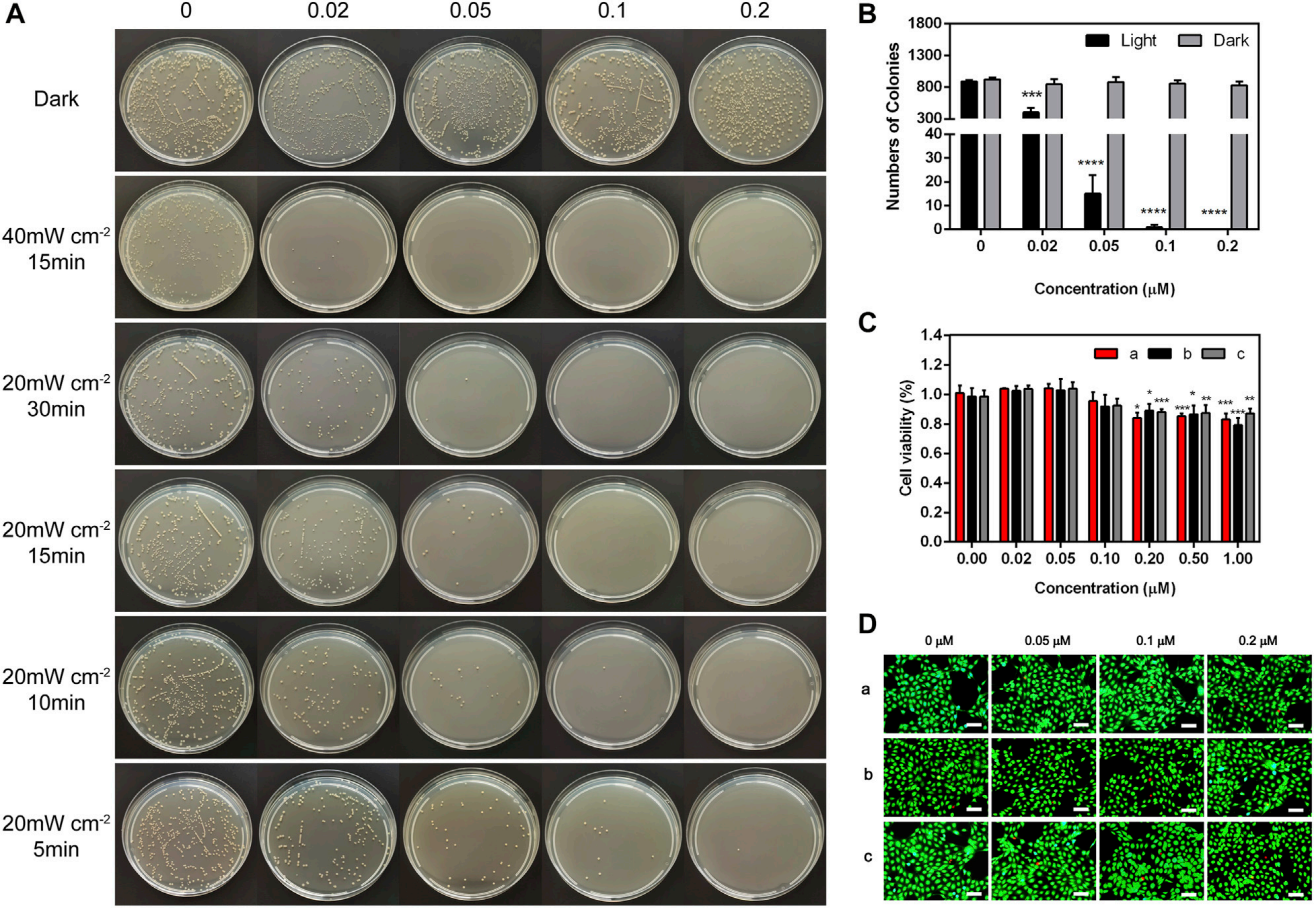
**(A)** Representative photographs of *S. aureus* treated by TTVP at different concentrations and irradiation conditions. **(B)** The surviving colony count of *S. aureus* treated by TTVP in light condition (20 mW/cm^2^) for 15 min. **(C,D)** Cell viability and Live/dead staining images of HCECs treated with TTVP, **(a)** with light after incubation with TTVP for 15 min and **(b)** without light after incubation with TTVP for 15 min **(c)** treated by TTVP for 24 h without light (scale bar: 100 μm). *for comparison among different concentrations of TTVP (0.02, 0.05, 0.1, 0.2, 0.5 and 1 μM) versus TTVP (0 μM) in light and dark conditions. *p < 0.05, **p < 0.01, ***p < 0.001 and ****p < 0.0001.

The antibacterial property *in vitro* was tested through traditional spread plate method, and the bacteriostasis was evaluated intuitively by comparing the number of viable bacteria (Zhang et al., 2022a). The bacterial growth in the plate was recorded and the antimicrobial rate of TTVP was calculated. First of all, as shown in Figure 2A, TTVP exerted negligible toxicity without light irradiation process after various concentrations of TTVP intervened. Next, the *S. aureus* incubated with TTVP was irradiated in an intensity of 40 mW/cm^2^ for 15 min, showing good bactericidal ability at the concentration of 0.02 μM. In order to optimize the illuminance, we halved the light intensity of 20 mW/cm^2^ and performed at different durations including 5, 10, 15 and 30 min. As shown in Figure 2A, the antibacterial effect of TTVP exhibited a dose-dependency feature, and enhanced gradually along with the increased concentrations ranging from 0 to 0.2 μM. Amazingly, *S. aureus* was killed effectively with irradiation (20 mW/cm^2^) for 15 min, along with 0.1 μM TTVP co-cultured (Figure 2B). In order to better bactericidal effect, this intervention method was applied for further experiments.

### 3.3 Antibacterial mechanisms of TTVP

**FIGURE 3:**
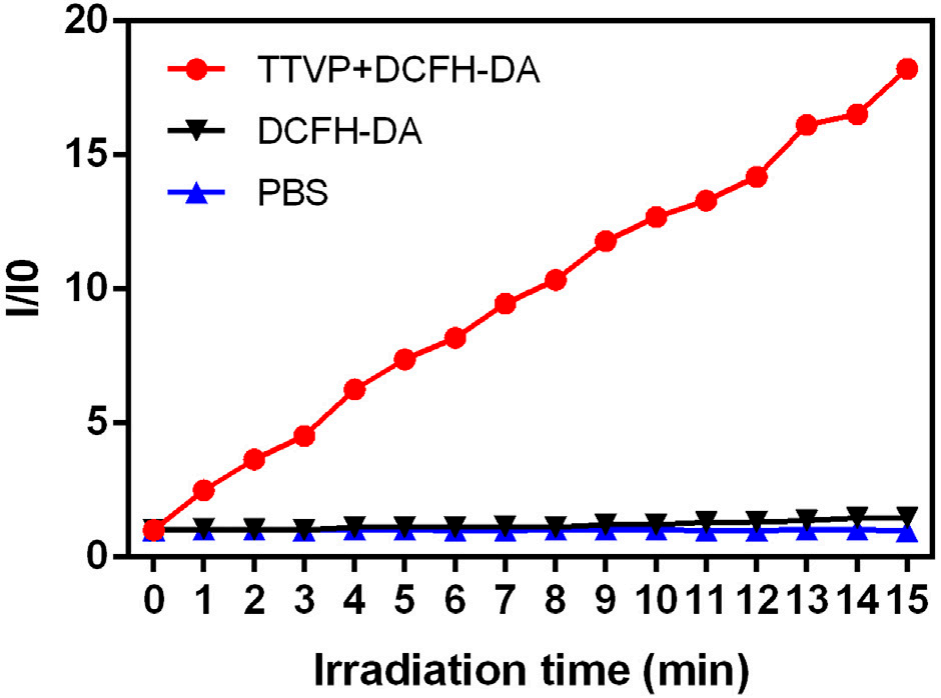
ROS generation of TTVP at 0.1 μM under light irradiation (20 mW/cm^2^) for 15 min.

Under light irradiation (20 mW/cm^2^), we further explored the mechanisms of TTVP killing *S. aureus* by measuring the ROS generation ability within 15 min. As shown in Figure 3, Supplementary Figure S2, abundant ROS was generated by TTVP at the concentration of 0.1 μM under white light irradiation, which demonstrated that AIEgens could generate efficient ROS in an aggregation state. The possible mechanism was the block of energy consumption by the way of non-radiative channels and aggregation-induced intersystem crossing (AI-ISC), inducing the transfer of facilitated energy state between singlet (S1) and triplet (T1) (Xu et al., 2015b; Yang et al., 2016). What’s more, our previous research has shown a terrific generation of singlet oxygen in the aggregation state, inducing the antibacterial ability of TTVP (Lee et al., 2020). Thus, TTVP was expected to be a meaningful photosensitizer for PDT evolvement.

### 3.4 Imaging of HCECs cells and *S. aureus* mixure

The zeta potential value of pure *S. aureus* was −25.31 ± 0.54 mV, while the values were increased to −21.76 ± 1.04, −21.06 ± 1.13, −20.71 ± 0.90 mV, and −18.99 ± 1.50 mV after incubated with TTVP (0.02, 0.05, 0.1 and 0.2 μM), which verified the affinity of TTVP to *S. aureus* (Supplementary Figure S3). The co-culture method of HCECs and *S. aureus* was further employed to verify the selective killing ability of TTVP. As shown in Supplementary Figure S4, in a co-culture system of HCECs and *S. aureus*, red fluorescence was observed in *S. aureus* (area circled) and extremely weak in HCECs. TTVP could bind *S. aureus* specifically under certain concentrations for the surface charge discrepancy. Such a high selectivity reduced the damage on the normal corneal cells, thus implying promising application in BK treatment.

### 3.5 Biocompatibility evaluation

The biocompatibility of AIEgens should be taken into serious consideration prior to biological application. Since TTVP was administrated on the cornea, HCECs were performed to evaluate the biosafety. In Figure 2C, no significant difference was observed in cell viability of HCECs incubated with TTVP at concentrations under 0.1 μM in dark and light condition. Meanwhile, the cellular morphology and density were observed visually by optical microscope. In Supplementary Figure S5, no obvious cell morphology change and density decrease were observed after TTVP incubation. What’s more, live/dead staining of HCECs was performed to evaluate the cytotoxicity of TTVP. As shown in Figure 2D, the results showed no meaningful differences between live and dead fluorescence after being treated with TTVP (0–0.2 μM) under dark or light condition (20 mW/cm^2^, 15 min), indicating low cytotoxicity of TTVP. In general, a hemolysis ratio no more than 10% was considered safe and acceptable for biological application (Xu et al., 2015a). It was found that the hemolysis rates were less than 1% after TTVP was added to blood, and calculated to be 0.27% ± 0.001% at the concentration of 0.2 μM (Supplementary Figure S6). Therefore, TTVP in our study was expected to be a promising option *in vitro* for further application.

### 3.6 *In vivo* treatment of BK

#### 3.6.1 Clinical observation

**FIGURE 4:**
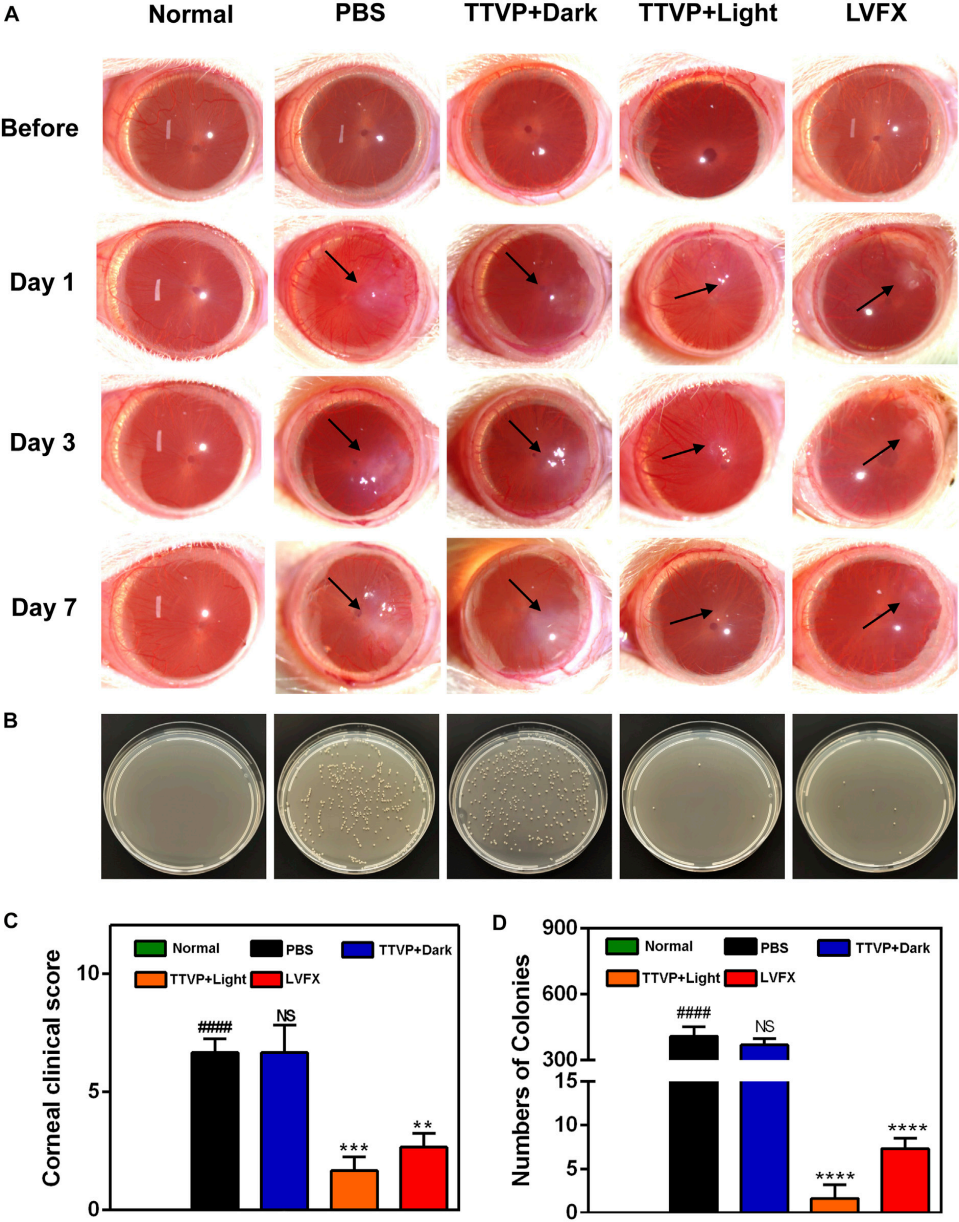
**(A)** Representative photographs of rat eyes before and at 1, 3 and 7 days after treatment. **(B)** The bacterial cultures on agar plates from the corresponding cornea. **(C)** Clinical score of rat eyes after different treatments; **(D)** the number of surviving *S. aureus* in corneas after different treatments at 7 days. # for group PBS *versus* group Normal, *for group TTVP + Dark, TTVP + Light, LVFX *versus* group PBS, *p or ^#^ *p* < 0.05, **p or ^##^ *p* < 0.01, ***p or ^###^ *p* < 0.001, ****p or ^####^ *p* < 0.0001, and NS for not significant.

Encouraged by the high antibacterial activity, we employed TTVP to treat severe BK, which is prone to relapse and often leads to refractory ocular damage and blindness. Based on the promising antibacterial efficacy and reliable security of TTVP, we established an experimental keratitis rat model to investigate whether TTVP could be applied for bacterial keratitis treatment. As shown in Figure 4A, after inoculation for 24 h, all rats appeared conjunctival congestion, corneal opacity and decreased corneal transparency to the same extent. Then, after different treatments, obvious differences in ocular manifestation were observed in these groups. In the PBS and TTVP under dark condition groups, the infection worsened severely over time. Three days after infection, the rat cornea exhibited prominent clinical manifestations of edema and muddiness at the damaged lesions. On the contrary, in TTVP with light group (20 mW/cm^2^ power, 15 min) and LVFX group, the severity of infection was well-controlled, while the TTVP under light condition exhibited better antibacterial efficacy. At day 7, the groups treated with PBS and TTVP without light irradiation showed severe bacterial infection, displaying a more pyknotic corneal opacity. Amazingly, the eye symptoms were alleviated and the transparency of the ocular surface was enhanced in the TTVP with light irradiation group, which was superior to the LVFX groups.

In Figure 4C, the groups treated by TTVP with light irradiation showed significantly lower clinical scores than dark or PBS groups. This demonstrates that light activation of TTVP could effectively reduce the degree of infection, which was consistent with the photographs of rats’ corneas. The strengthened antibacterial ability may be attributed to the ROS potency after light irradiation.

In addition, the corneal tissues were isolated from each group, and then homogenized and distributed on the spread plate to quantitatively evaluate the surviving bacterial colonies. The plates displayed nearly no colony formation in TTVP with light irradiation group, which showed better therapeutic effects than LVFX, further indicating the remarkable therapeutic efficacy of TTVP against BK (Figures 4B, D). Due to the incidence of bacteria resistance after antibiotics application, the sterilization process of TTVP under light condition showed great potential for the treatment of *S. aureus*-infected keratitis.

#### 3.6.2 Histopathological analysis

**FIGURE 5:**
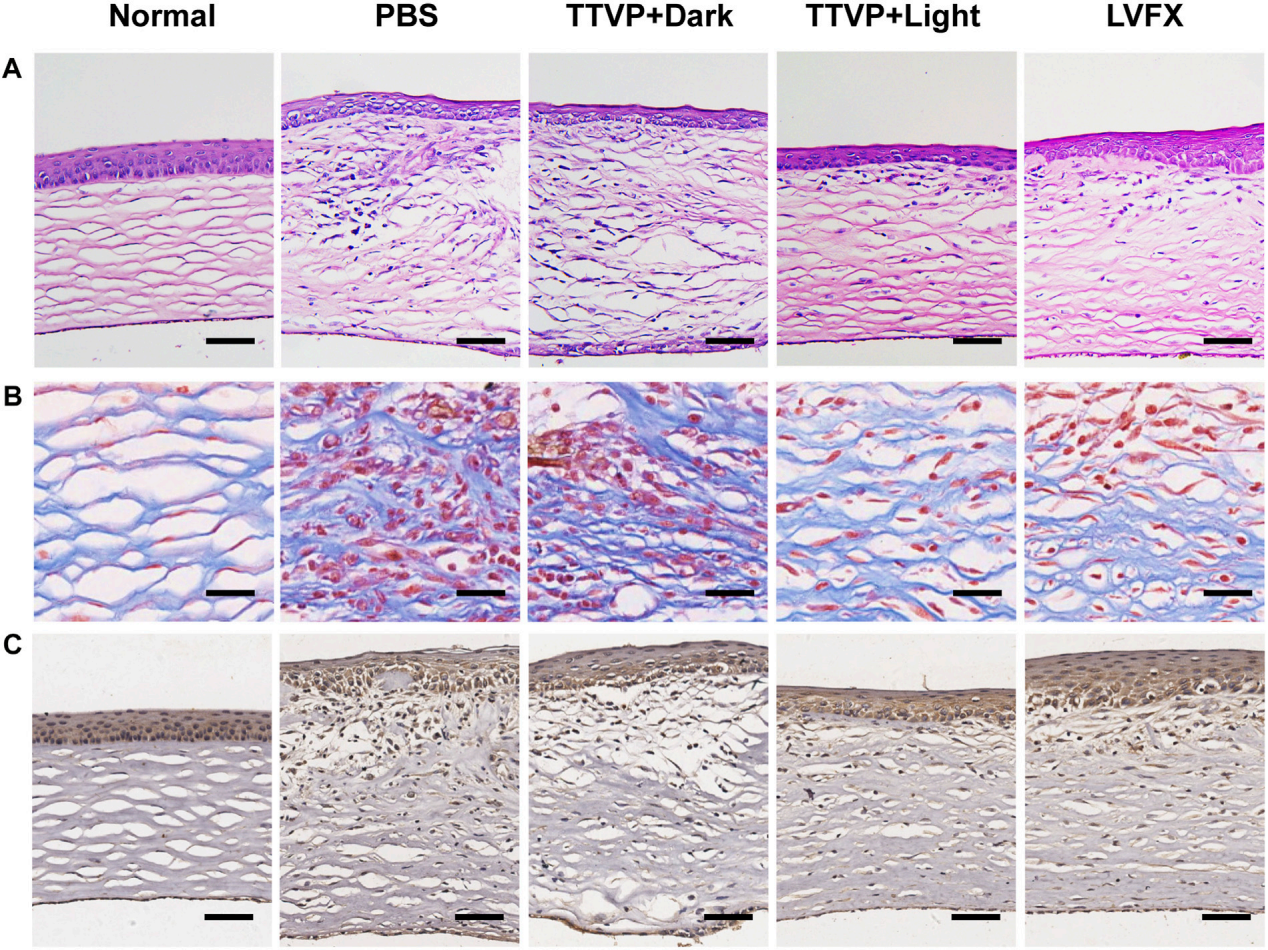
**(A)** H&E staining (scale bar: 100 μm), **(B)** Masson staining (scale bar: 25 μm), and **(C)** Immunohistochemistry staining of TNF-α (scale bar: 100 μm) of cornea in different groups.

Figure 5 displayed photomicrographs of corneal tissue sections stained with H&E, Masson and immunohistochemistry staining of different groups. The normal cornea is composed of five layers, with continuous corneal epithelium and endothelial layer, as well as stromal connective tissue layer with no inflammatory-cell infiltration (Bharti and Kesavan, 2017). Masson staining is used to display the fibers in the tissue and visualize the structure of collagen in the stromal tissue (Meng et al., 2019). However, in PBS and TTVP without light irradiation groups, the epithelial layer was dense and stratified, the stromal layer was apparent edema and massive inflammatory infiltration, and the endothelial layer was focally dense. In comparison with these two groups, TTVP under light irradiation group revealed less severity of corneal edema and reduced inflammatory cell infiltration, which could be attributed to rapid sterilization *via* ROS generation to alleviate inflammatory response. And the groups treated with LVFX showed less antibacterial ability than TTVP with light irradiation. Next, immunohistochemistry staining of TNF-α was performed to assess the therapeutic efficacy, which was a vital index to evaluate the Inflammation levels. The results showed that the cornea treated with TTVP under light condition exhibited a significant reduction in inflammation levels. All the histopathological results were consistent with the ocular manifestations.

### 3.7 *In vivo* biosafety

**FIGURE 6:**
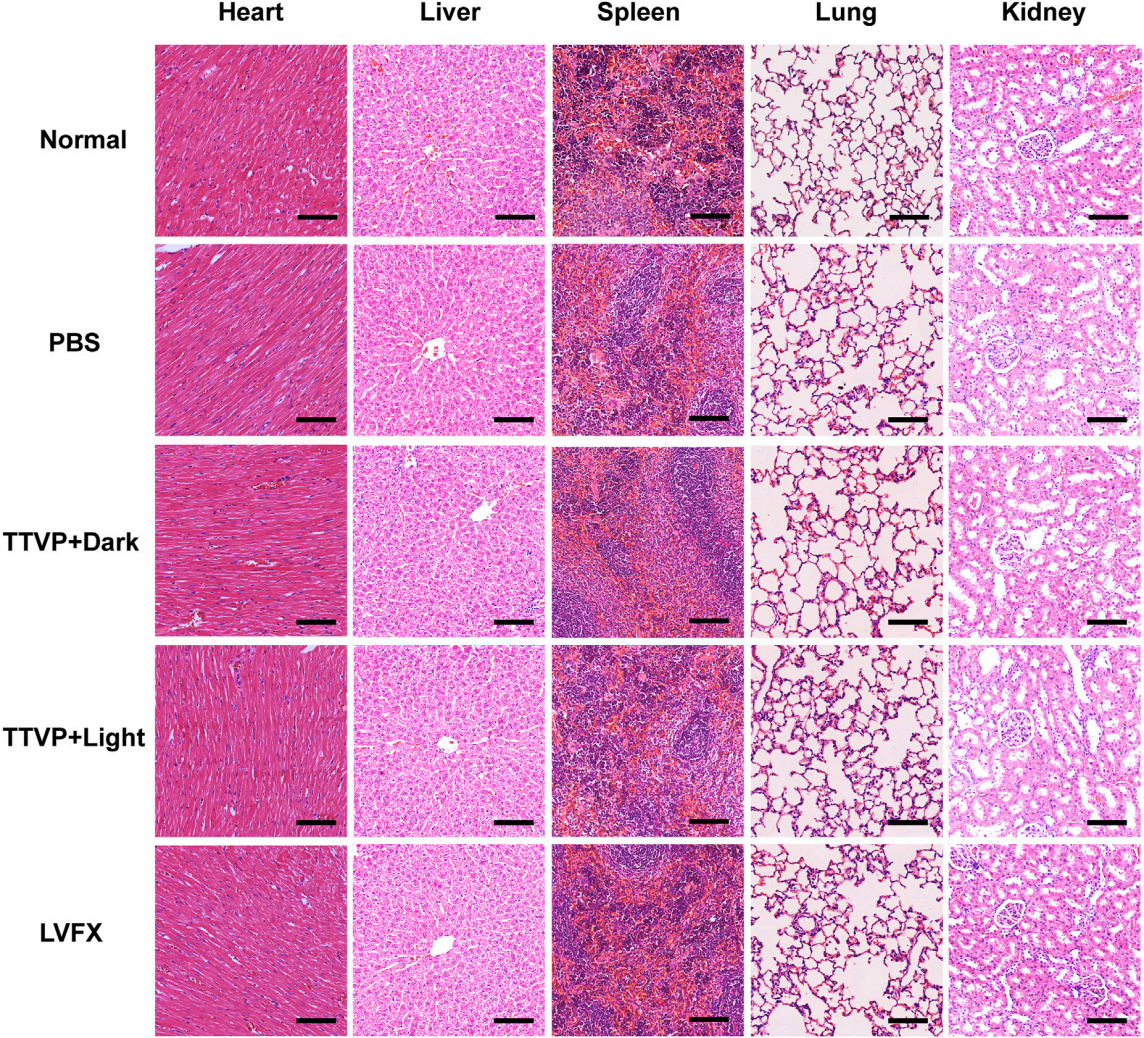
H&E staining of main visceral organs at 7 days post treatment (heart, liver, spleen, lung and kidney, scale bar: 100 μm).

**FIGURE 7:**
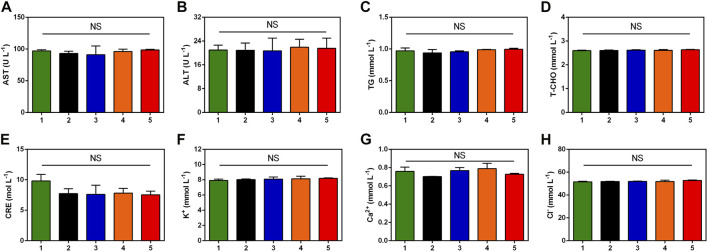
Blood biochemistry examination analysis of rats after different treatments. **(A)** AST **(B)** ALT **(C)** TG **(D)** T-CHO **(E)** CRE **(F)** K^+^ **(G)** Ca^2+^ **(H)** Cl^−^. Group Normal (1), Group PBS (2), Group TTVP + Dark (3), Group TTVP + Light (4) and Group LVFX (5) (NS, not significant).

In order to assess the biosafety of TTVP *in vivo*, H&E staining was conducted on heart, liver, spleen, lung and kidney to detect the structural damage, while several biochemical indexes were applied to test the major functions of organs. H&E staining and biochemical indexes are extensively used to visually appraise the structural and functional change respectively (Abdelhalim et al., 2018; Zhang et al., 2018; Nguyen et al., 2019). As shown in Figure 6, no histomorphological changes were revealed in main organs *in vivo*, which proved the safety of TTVP in organ structure. Meanwhile, no obvious discrepancy was shown in the content of AST, ALT, TG, T-CHO, CRE, K^+^, Ca^2+^ and Cl^−^ among all groups, which indicated excellent liver and renal functions, lipid levels, and electrolyte stabilization in this study (Figure 7). TTVP was thus expected to be a promising option *in vivo* for BK application.

## 4 Conclusion

To sum up, a TTVP-mediated and ROS-induced PDT therapy was estimable in bactericidal research of BK. TTVP displayed low toxicity and excellent biocompatibility, ensuring further biological applications. TTVP could selectively combine with *S. aureus* over corneal cells. TTVP possessed excellent ROS release capacity, and showed specific antibacterial function without apparent cytotoxicity. TTVP exhibited vital status of photodynamic antibacterial therapy over BK injury. Our findings demonstrated that TTVP showed promising possibility as antimicrobials, which may take advantage of clinical application of BK treatment. This work will stimulate extensive application of TTVP as a novel therapy against BK infection.

## Data Availability

The original contributions presented in the study are included in the article/Supplementary Material, further inquiries can be directed to the corresponding authors.
