# Supplementary material for: Efficient antibacterial AIEgens induced ROS for selective photodynamic treatment of bacterial keratitis
Source: Front Chem. 2023 Jan 4;10:1088935. doi: 10.3389/fchem.2022.1088935 (PMC9846558; doi:10.3389/fchem.2022.1088935)
Supplement: Supplementary file 1 [file DataSheet1.docx]

**Supplementary figures**

**Efficient Antibacterial AIEgens Induced ROS for Selective Photodynamic Treatment of Bacterial Keratitis**

Wenting Cai^1,†^, Tianyi Shen^1,†^, Dong Wang^4^, Tingting Li^1^, Jing Yu^1^, Chen Peng^1,3,*^, Ben Zhong Tang^2,4,*^

^1^Department of Ophthalmology, Shanghai Tenth People’s Hospital, School of Medicine, Tongji University, Shanghai 200072, China

^2^Shenzhen Institute of Molecular Aggregate Science and Engineering, School of Science and Engineering, The Chinese University of Hong Kong, Shenzhen 518172, China

^3^Department of Radiology, Shanghai Public Health Clinical Center, Fudan University, Shanghai 201508, China

^4^College of Materials Science and Engineering, Shenzhen University, Shenzhen 518060, China

^†^These authors have contributed equally to this work


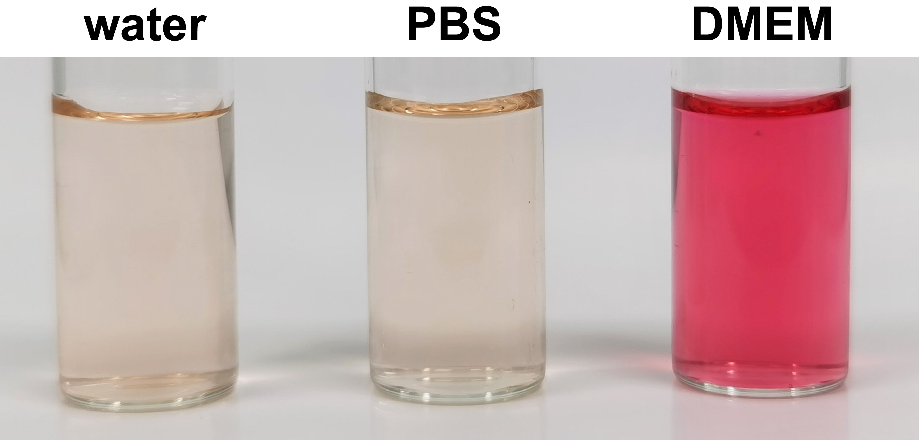


**Figure S1.** The photograph of TTVP solution (5 μM) diluted with water, PBS and DMEM, respectively.


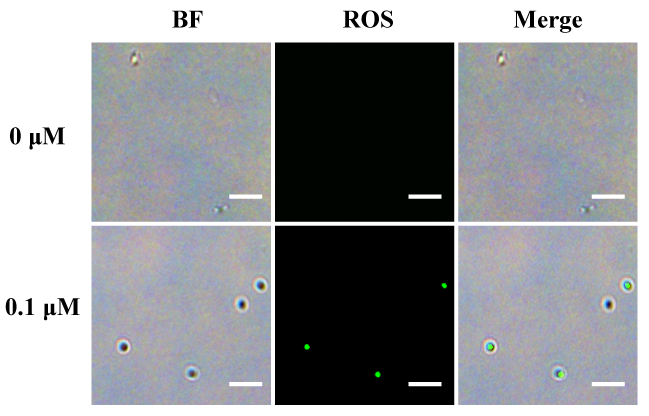


**Figure S2.** ROS production of TTVP after light irradiation via fluorescence microscope (scale bar: 25 μm)





**Figure S3.** Zeta potentials of *S. aureus* incubated with different concentrations of TTVP for 15 min *(** P < 0.01)*


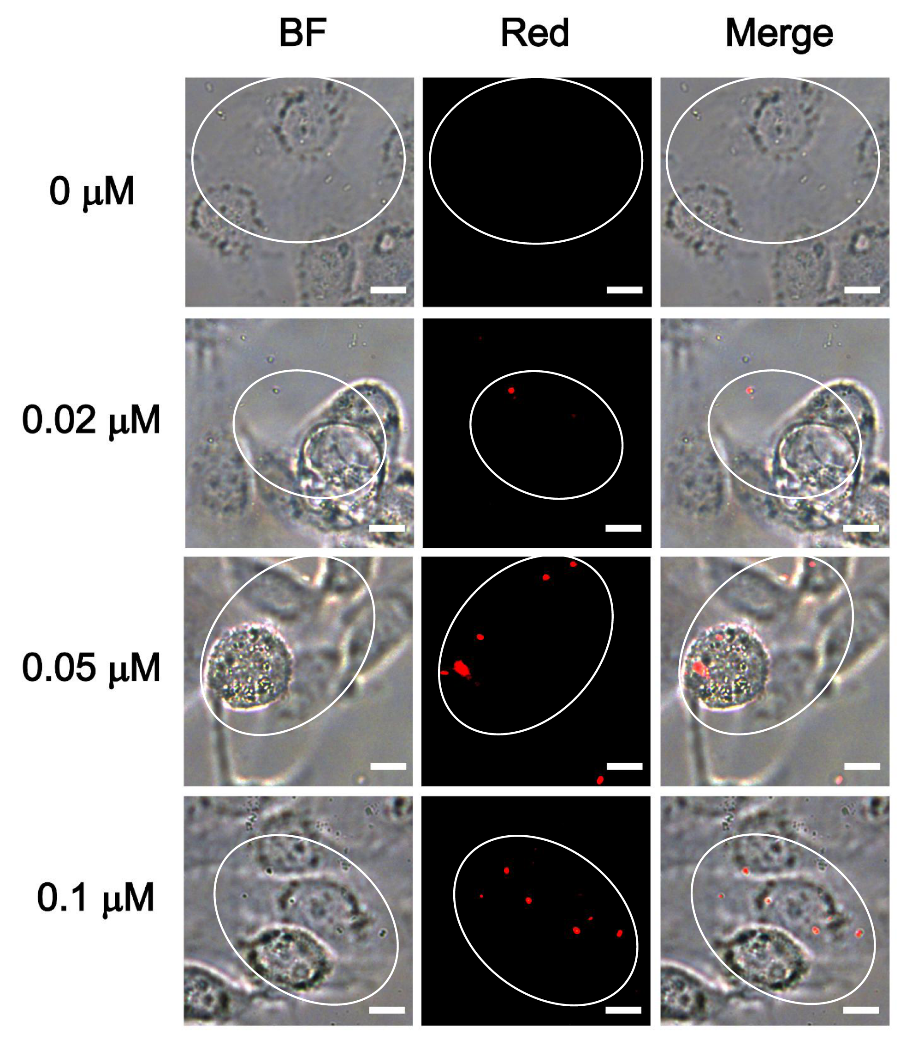


**Figure S4.** Fluorescence images of a mixed suspension containing HCECs and *S. aureus* incubated with TTVP (scale bar: 10 μm)


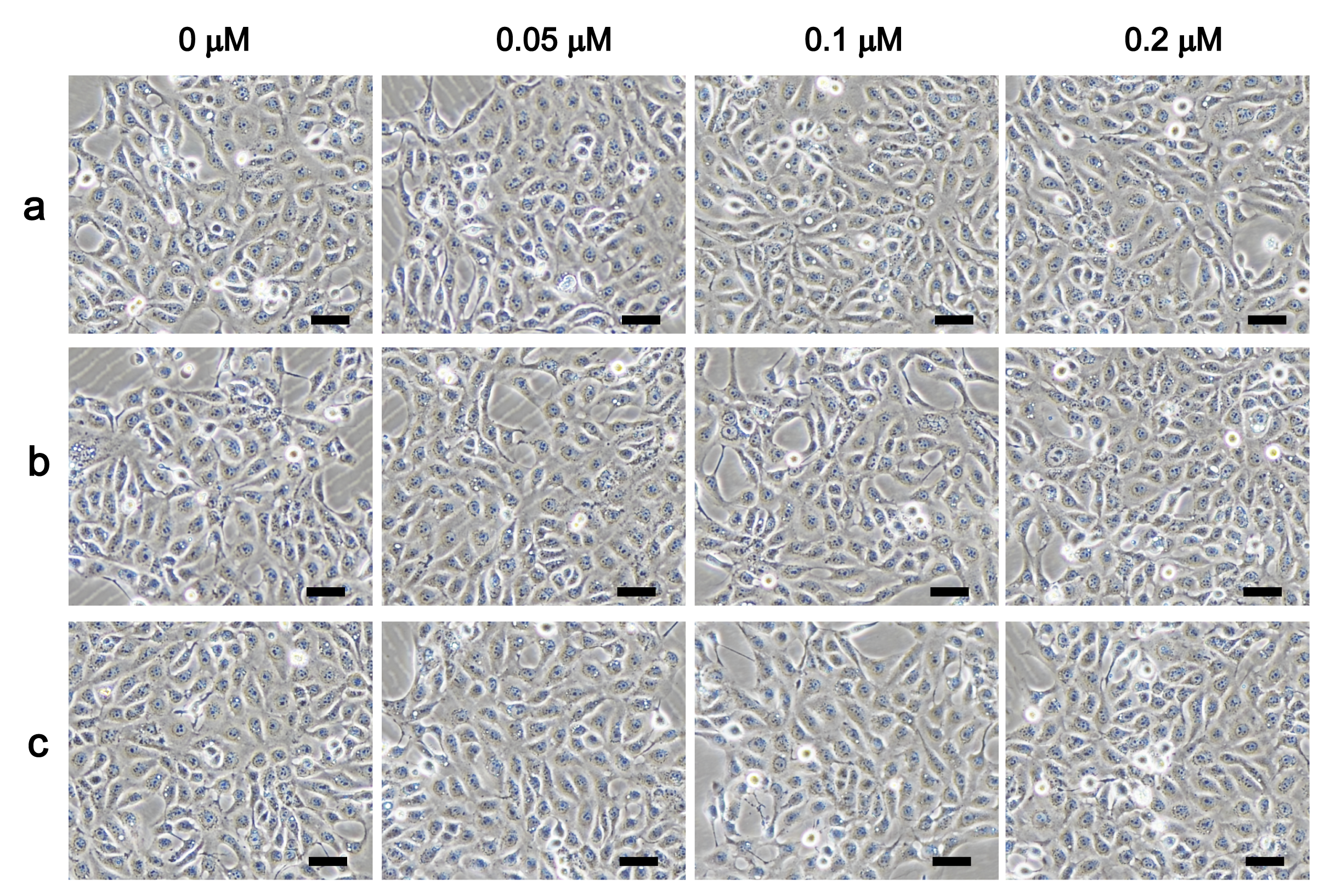


**Figure S5.** Cell morphology of HCECs incubated with different concentrations of TTVP in different irradiation conditions. (a) and (b) treated with and without light after incubation with TTVP for 15 min, respectively; (c) treated by TTVP for 24 h without light (scale bar: 100 μm).


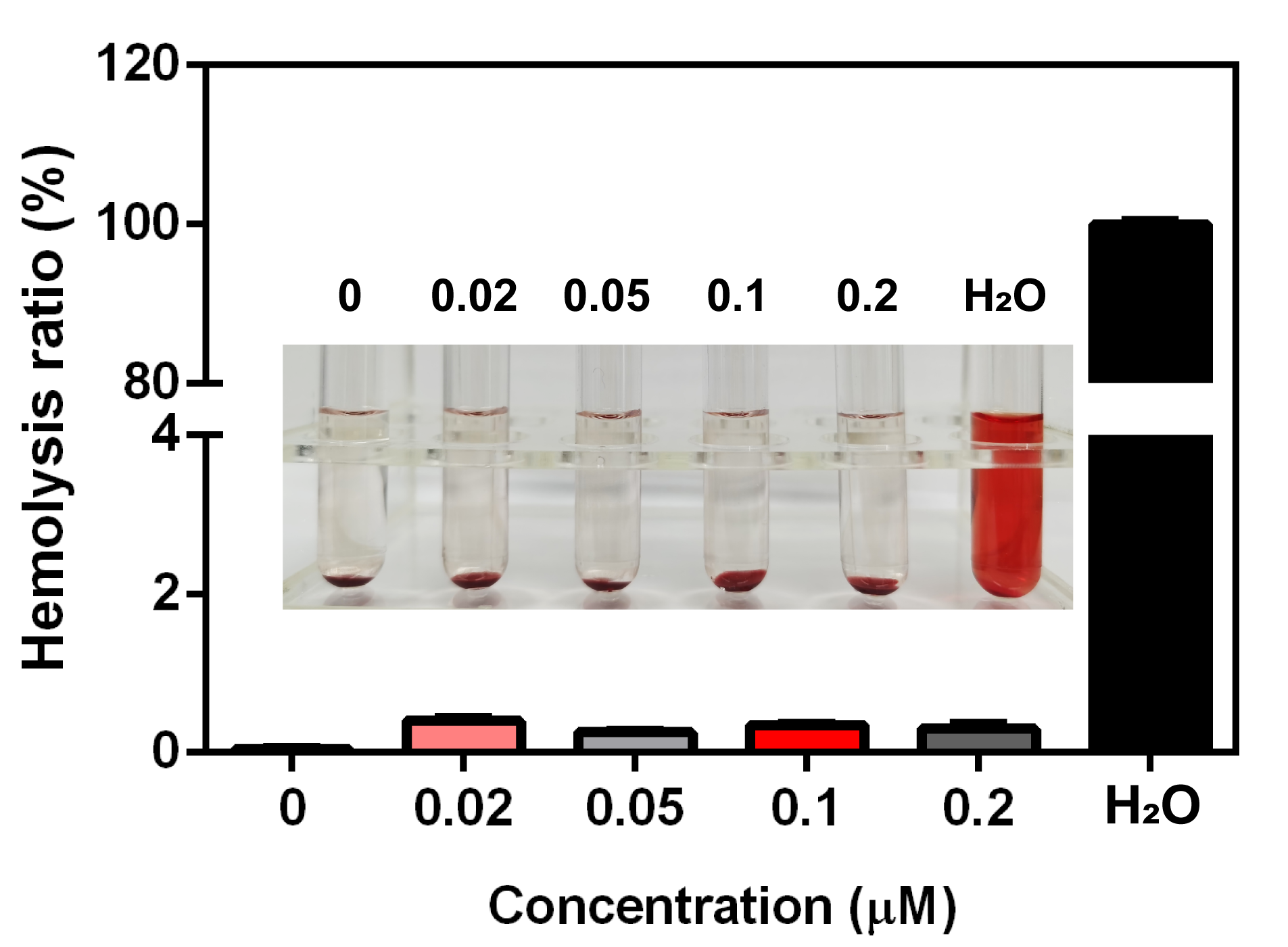


**Figure S6.** Hemolysis assay of erythrocytes treated with TTVP at different concentrations.
